# Density estimates reveal that fragmented landscapes provide important habitat for conserving an endangered mesopredator, the spotted-tailed quoll

**DOI:** 10.1038/s41598-022-16982-x

**Published:** 2022-07-25

**Authors:** T. Henderson, B. A. Fancourt, R. Rajaratnam, K. Vernes, G. Ballard

**Affiliations:** 1 Ecosystem Management, School of Environmental and Rural Science, University of New England, Armidale, NSW 2351 Australia; 2 Kangaroo Island Landscape Board, Kingscote, SA 5223 Australia; 3 Geography and Planning, School of Humanities and Social Science, University of New England, Armidale, NSW 2351 Australia; 4 Vertebrate Pest Research Unit, Department of Primary Industries, University of New England, Building C02, Armidale, NSW 2351 Australia

**Keywords:** Environmental impact, Conservation biology, Ecological modelling, Population dynamics, Ecology, Ecology, Environmental sciences

## Abstract

Native predators are increasingly exposed to habitat loss and fragmentation globally. When developing conservation and management strategies, it is important to determine whether fragmented landscapes can still support similar predator densities to intact areas, and thereby constitute important habitat for these species. The spotted-tailed quoll (*Dasyurus maculatus*) is an endangered Australian mesopredator that is often considered to be forest-dependent. While quolls are known to occur in some fragmented forest landscapes, it is unclear whether these areas represent sub-optimal habitat where quolls merely persist, or whether quolls can still occur at densities similar to those observed in intact forest landscapes. We used camera traps to detect quolls in both a fragmented and intact forested site, over three years. We used each quoll’s unique pelage pattern to identify individual quolls and estimate population density at each site. We were able to assign more than 94% of quoll image sequences across both sites to identify 173 individuals during the study. Density estimates of 0.13–0.66 quolls per km^2^ at the fragmented site were comparable to estimates of 0.28–0.48 quolls per km^2^ at the intact site. Our results highlight the importance of retaining and protecting forest fragments for the conservation of endangered quoll populations.

## Introduction

Habitat loss and fragmentation are the main causes of biodiversity decline around the world, as humans continue to modify and clear natural habitats^1–3^. Native predators are increasingly threatened by habitat fragmentation^4,5^ and associated impacts of reduced habitat resources and increased conflict with other predators and humans^6–8^. For example, in Madagascar, small native predators such as spotted fanaloka (*Fossa fossana*) and ring-tailed vontsira (*Galidia elegans*) have suffered range contractions due to habitat loss, hunting by humans, and competition with introduced predators^8,9^. However, some native mesopredators are known to persist and even thrive in fragmented landscapes^4,10^. For example, pine martens (*Martes martes*) occur in fragmented landscapes in Europe, utilising supplementary prey and habitat resources^11,12^. Similarly, güiña (*Leopardus guigna*) occupy forest fragments and edge habitats in fragmented landscapes in Chile^13,14^. As native predators continue to be impacted by habitat loss, a key challenge is understanding their ecology in fragmented landscapes to inform conservation and management of these species.

The spotted-tailed quoll (*Dasyurus maculatus*) is a medium-sized (males 2.0–4.2 kg; females 1.2–2.1 kg^15^) endangered mesopredator endemic to Australia. Since European colonization, the spotted-tailed quoll (hereafter referred to as ‘quoll’) has suffered a 50–90% decline in its range^16^, primarily due to habitat loss and associated increase in competitive interactions with introduced predators such as red foxes (*Vulpes vulpes*)^17,18^. On mainland Australia, quolls have predominately been studied in large, intact, forested landscapes^19–21^. They are considered a forest-dependent species^18,22^, presumably because forested habitats are thought to provide quolls with refugia from foxes that are typically more prevalent in fragmented and open agricultural landscapes. However, previous research suggests that quolls can persist in fragmented habitats on mainland Australia^23^ as well as in the island state of Tasmania ^24–26^. While foxes are absent from Tasmania, they are widespread across most of mainland Australia, where quoll ecology in fragmented landscapes is poorly understood. To inform meaningful conservation strategies, it is important to understand to what degree quoll populations persist in fragmented landscapes.

When developing conservation and management strategies for animal populations, an estimate of a species population size if often required^27^. For species with distinct markings, such as the quoll, spatial capture-recapture (SCR) models can be used to estimate population density. SCR models are an extension of conventional capture-recapture models that incorporate the spatial distribution and movements of known individuals relative to capture locations^28^. SCR models allow for more flexible study designs as they eliminate the need for estimating the area effectively sampled^28^, which is useful when attempting to estimate density of rare or cryptic carnivore species that occur across large areas^29^. For example, SCR has been used to estimate densities of tigers (*Panthera tigris*) and leopards (*Panthera pardus*) in India^30^, as well as brown hyenas (*Parahyaena brunnea*) and spotted hyenas (*Crocuta crocuta*) in Botswana^31^.

In this study, we investigated whether quolls in a fragmented forested site on mainland Australia persisted at relatively low densities, or whether they persisted at similar densities to populations in a nearby intact forested site. We used camera traps to detect quolls over a three-year period and utilised their uniquely spotted pelage to identify individuals at each site. The detection and spatial re-detection of individual quolls was then used to estimate quoll density at each site using SCR models. We predicted that the fragmented site would support lower densities of quolls than the intact site, due to anticipated negative impacts from habitat fragmentation.

## Methods

### Study sites

**Figure 1:**
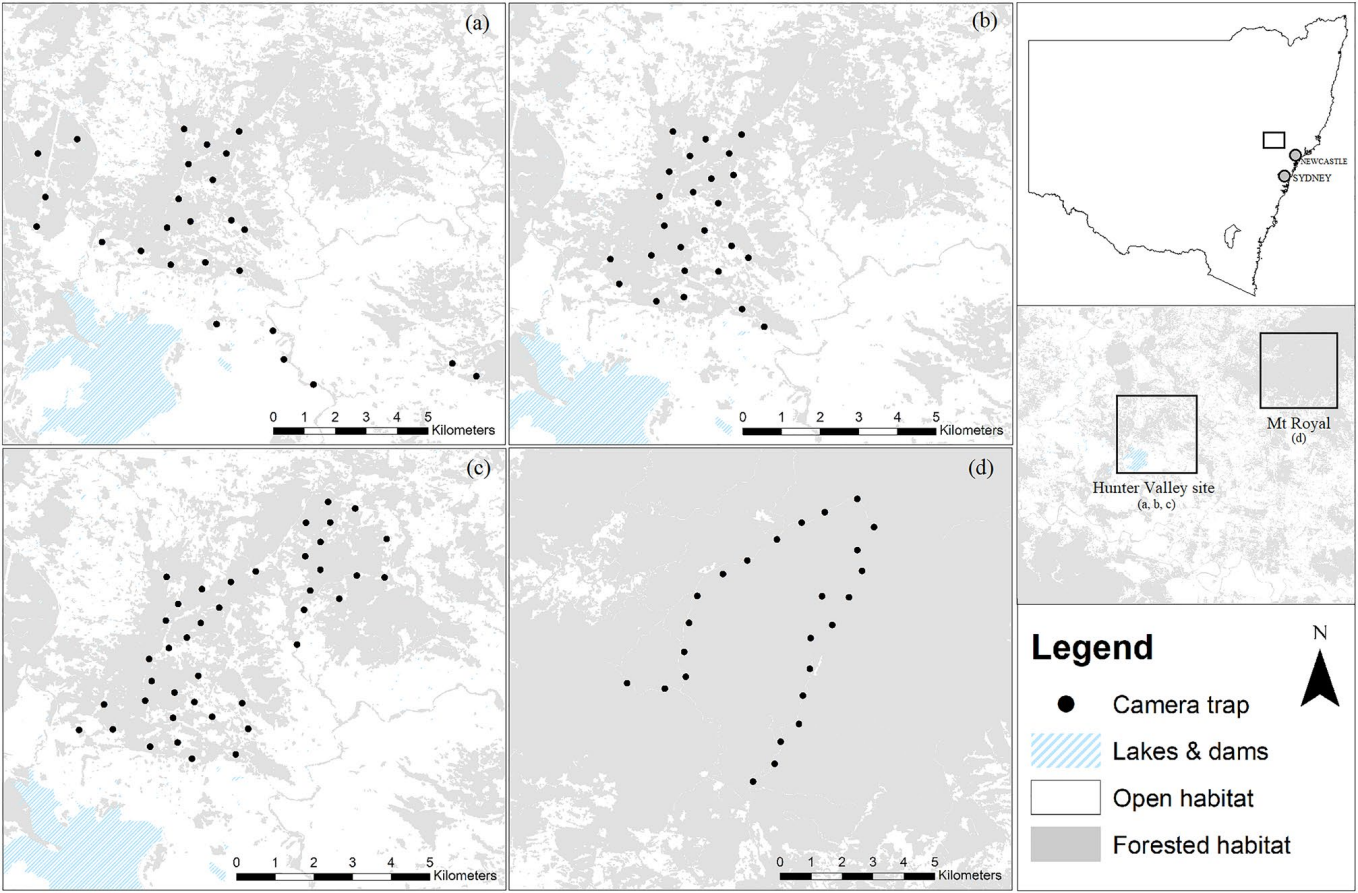
Study site locations for estimating spotted-tailed quoll (*Dasyurus maculatus*) densities in fragmented and intact landscapes within the Hunter region in New South Wales, Australia. Figures show the camera trap layout for the Fragmented site Study 1 (**a**), Study 2 (**b**), Study 3 (**c**), and the Intact site (**d**). This figure was created using ArcGIS version 10.4.1 (www.esri.com).

Camera trapping was conducted across two sites in the Hunter Region of New South Wales, Australia (Fig. 1). The first site was located in the Hunter Valley in a fragmented forest landscape not associated with any large, contiguous forest. The site comprised remnant sclerophyll forest, grassy woodland, and small pockets of dry rainforest intermixed with open areas cleared for mining and agriculture. Data for this site came from three different camera trap studies, each with different camera trap layouts and survey effort (Fig. 1a–c). The second site was located within Mt Royal National Park, which comprised an intact, contiguous forested landscape with a mix of sclerophyll forests and dry rainforest, located approximately 20 km north-east of the Hunter Valley site. Data for this site came from a single camera trap study (Fig. 1d).

### Study design

At the Hunter Valley (‘Fragmented’) site, ‘Study 1’ was conducted from July 2018 to October 2019 and consisted of 25 randomly allocated camera trap locations (Fig. 1a) as described in Henderson et al.^23^. For ‘Study 2’, the 25 camera traps were condensed into a more focal study area (Fig. 1b) and surveys were conducted from October 2019 to May 2020, as described in Henderson, et al.^32^. For ‘Study 3’, an expanded study area was used which consisted of 42 camera trap locations (Fig. 1c; Henderson et al. (in prep)), with surveys conducted from May 2020 to December 2020. At the Mt Royal National Park (‘Intact’) site, 25 cameras traps were deployed continuously from August 2019 to August 2021 (Fig. 1d) as described in Henderson et al.^32^. For each study, camera traps were spaced at least 500 m from adjacent cameras, to approximate the minimum home range of female quolls^19^. Fine-scale camera trap locations were selected based on the presence of suitable habitat features such as fallen logs required for camera trap setup. Research at these sites were approved by the Animal Ethics Committee at the University of New England, Australia.

Reconyx HC600 infrared cameras (Reconyx, Holmen, USA) were used for all surveys. Each camera was positioned ~ 1.0 m above the ground and attached to a metal post which faced a large horizontal log located between 1.5 and 3.0 m in front of the camera. A vented lure canister containing ~ 500 g of raw chicken necks was pegged into the ground in front of the log. To increase the probability of quoll detections, cameras were positioned so that the camera’s upper detection zone covered the top of the log and the lower detection zone covered the lure canister^23^. All cameras were programmed to take a rapid-fire sequence of 10 images per motion trigger on high sensitivity with no delay between triggers. Camera traps were serviced every 2–4 months, which included replacement of batteries, SD cards and lures, as well as checking camera alignment and functionality. The large and inconsistent variation in time between camera servicing across surveys was due to logistical and environmental constraints throughout the study.

### Individual quoll identification

**Figure 2:**
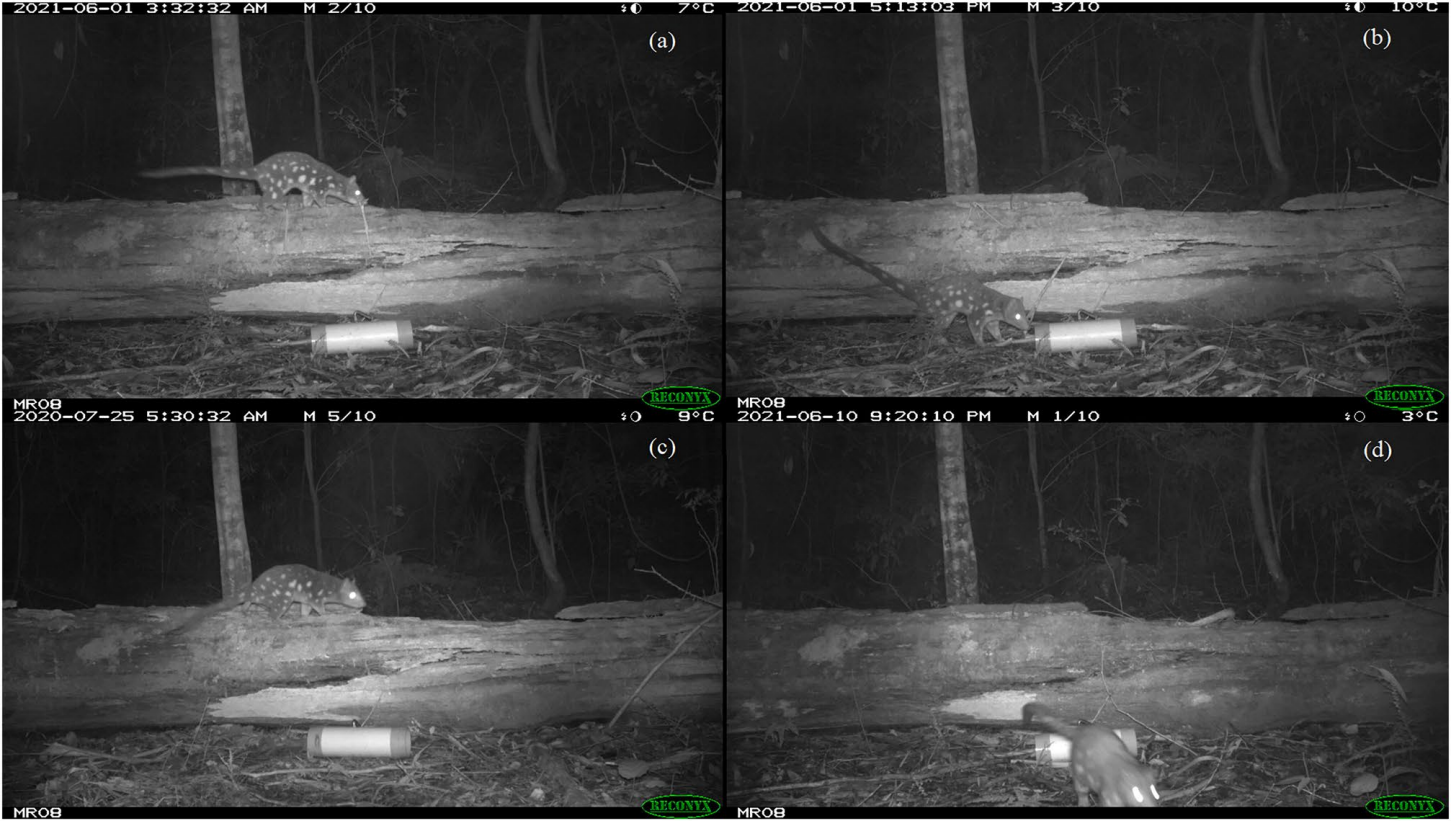
Example camera trap images of spotted-tailed quolls (*Dasyurus maculatus*) illustrating how images were classified. Images (**a**) and (**b**) show the right lateral side of the same individual quoll from different detection events. Image (**c**) is from a different individual, while image (**d**) would be tagged as ‘non-identifiable’.

To identify individual quolls, we assessed each quoll image sequence using the quoll’s unique spot patterns (Fig. 2). A catalogue of known individual quolls was progressively developed using a decision matrix flowchart (Supplementary Fig. S1). An individual quoll profile was defined as ‘complete’ if clear images of both the left and right lateral sides were obtained and did not match any previously profiled quolls. Individual quoll profiles were defined as ‘semi-complete’ if clear images of only one lateral side were obtained (either left or right; Fig. 2a–c) and did not match any previously profiled quolls (from either complete or semi-complete quoll profiles). Sequences of quoll images with unclear lateral spot patterns were categorised as ‘non-identifiable’. This included images that were blurry, obstructed, or only showed a small part of the quoll such as the tail, head or legs (Fig. 2d). While all images (n = 10) in each image sequence were assessed, often only one clear image was required for successful identification. All images in that sequence were assigned the same identification tag, as well as any image sequences that immediately followed (i.e. < 1 s between successive image sequences). In addition to spot patterns, other features such as size, shape, sex, or markings such as bald spots, injuries, or other pelage variations also assisted with identification of individuals e.g. Gorta et al.^33^. Quoll images were reviewed and tagged using Exifpro^34^ and were subsequently sorted into individual folders for each camera site, survey period, and study. For quoll profiles that were semi-complete, we only included the profiles from one lateral side (left or right) in subsequent analyses. At the Fragmented site, quolls with left-side only profiles were more frequent and therefore included. At the Intact site, quolls with right-side only profiles were more frequent and therefore included.

### Data analyses

#### Quoll detection events

For all profiled quolls, consecutive detections on the same camera were considered independent detection events if image sequences were separated by more than 10 min^23^. To standardise survey length across datasets, we first separated each study into distinct survey periods, with each new survey period commencing from the date when camera traps were serviced and lures refreshed. This was to ensure that the potential effect of lure age on quoll detectability remained consistent across survey periods^32^. This resulted in 12 surveys across the three studies at the Fragmented site, and seven surveys at the Intact site. As survey periods varied in duration, we adopted a standardised survey duration by truncating each survey back to the first 42 nights to match the duration of the shortest survey. We then calculated cumulative quoll detections and individual quolls for each night, for each survey period. Cumulative detections and individuals were then converted into a percentage of the total of number of detections and individuals for that survey. The mean proportion of cumulative detections and individual quolls for each night was calculated for each site. To help visualise quoll detections at each camera location for each study, we calculated the number of independent quoll detections per 100 camera trap nights.

#### Estimated quoll density

To estimate quoll density for each survey period at each site, we used the ‘secr’ package version 4.5.3^35^ in R version 4.2.0^36^. For SCR models, three data inputs are needed: (1) the total number of identifiable individuals encountered in each survey period; (2) the total number of re-encounters (to provide information on the baseline encounter rate); and (3) spatial re-encounters (to provide information on the movement parameter)^37^. SCR uses spatial detection histories to model the movement and distribution of individuals in space relative to the camera trap array^38^. We created spatial detection histories of individual quolls for each survey period using the R package ‘camtrapR’ version 2.0.3^39^. Each survey ran for 42 consecutive nightly occasions, with each occasion lasting 24 h (from 12:00:00 to 11:59:59 the following day). For all SCR models, we used the default ‘half-normal’ detection function^37^ with a buffer width of 4*σ* as recommended by Efford^35^. Each model included three parameters: *D,* animal density; *g*0, baseline encounter probability (probability of capture when the animals home range is centred on the camera); and *σ*, the spatial detection parameter. We investigated time and behavioural effects on the baseline encounter probability *g*0, where *D* and *σ* were kept constant (Table 1). These models tested several hypotheses: that the encounter probability changes based on quoll behaviour (1) after being detected (learned response), (2) after being detected at a specific site (site-specific response), or (3) linearly with time. We then ranked each model using Akaike Information Criterion values adjusted for small sample sizes (AICc)^40^ to determine model parsimony.

**Table 1:** Variables used in models to investigate their effect on the baseline encounter probability *g*0, while *D* and *σ* were held constant. Variable codes match code used in the R package ‘secr’.

Variable	Description
b	Learned response—behavioural change in encounter probability if individual had previously been detected
bk	Site-specific learned response—behavioural change (b) is specific to the camera location
B	Transient response—behavioural change in encounter probability, but only considers the previous occasion the individual was encountered
Bk	Site-specific transient response—behavioural change (B) is specific to the camera location
T	Time—encounter probability changes linearly with time
0	Null model—encounter probability is held constant

## Results

### Individual quoll identification

At both sites, over 94% of quoll image sequences were successfully assigned to a complete quoll profile (Table 2). Very few image sequences (≤ 1.3%) were assigned to semi-complete quoll profiles of only one lateral side (Table 2). Image sequences which could not be assigned to a profiled quoll (either complete or semi-complete) ranged from 3.1 to 5.0% across studies (Table 2).

**Table 2:** Camera trap effort, number of quoll image sequences, and the proportion of spotted-tailed quoll (*Dasyurus maculatus*) image sequences which were either assigned to a complete profiled quoll, a semi-complete profiled quoll (one lateral side only), or unable to be assigned to a profiled quoll (non-identifiable).

Site/study	Total effort (trap nights)	No. of image sequences	No. of sequences assigned to a profiled quoll (%)	No. of sequences assigned to a semi-profiled quoll (%)	No. of sequences not assigned to a profiled quoll (%)
Fragmented Study 1	11,509	2196	2112 (96.2%)	13 (0.6%)	71 (3.2%)
Fragmented Study 2	4883	440	418 (95.0%)	0 (0.0%)	22 (5.0%)
Fragmented Study 3	10,067	1294	1241 (95.9%)	13 (1.0%)	40 (3.1%)
Intact	17,690	3285	3098 (94.3%)	43 (1.3%)	144 (4.4%)

At the Fragmented site, 88 individual quolls were detected and identified across the three study periods, of which, 80% were detected on at least a second independent occasion (Supplementary Fig. S2). At the Intact site, there were 85 individual quolls detected, of which, 78% were detected on a second independent occasion (Supplementary Fig. S3).

### Quoll detection events

**Figure 3:**
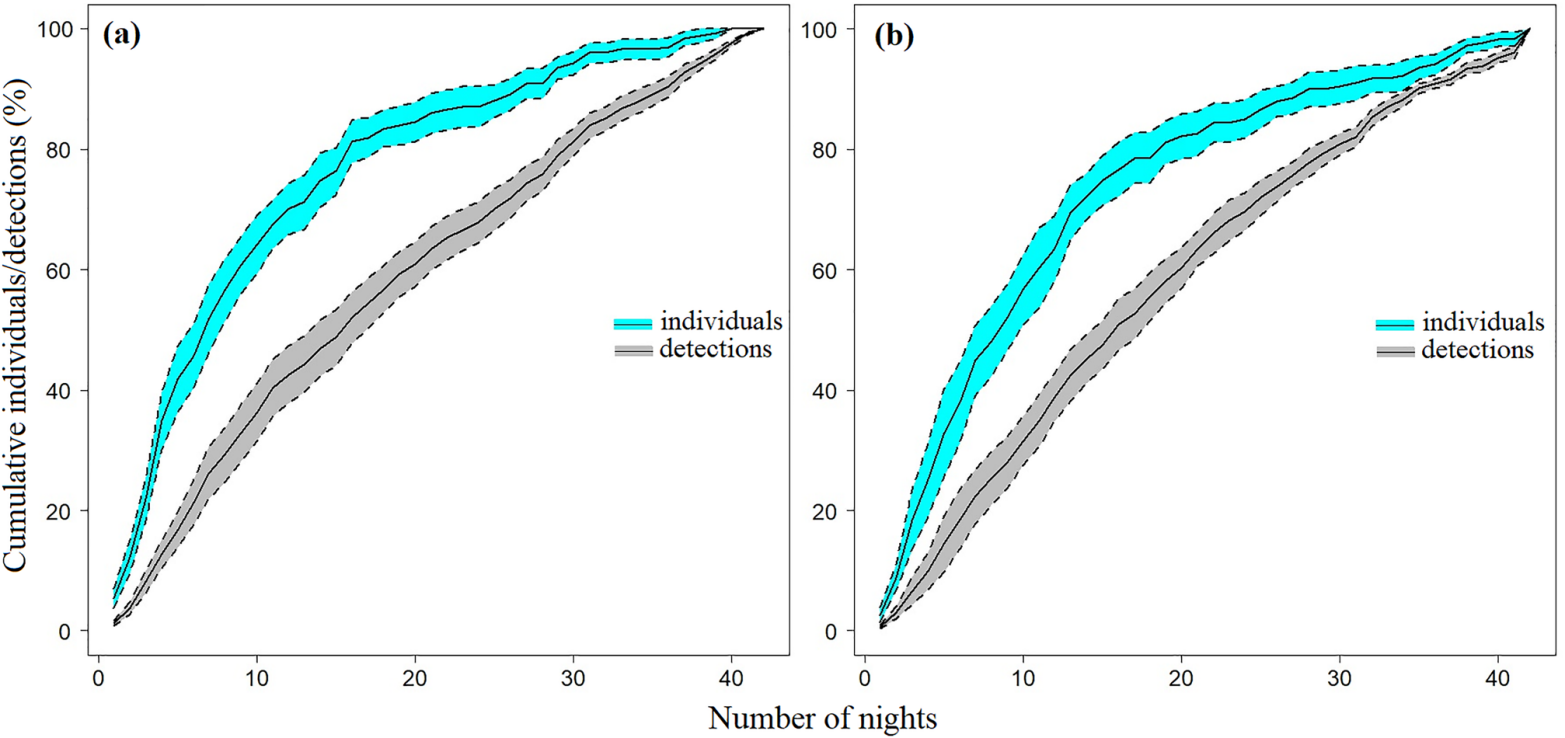
The mean cumulative proportion (%) of individual quolls (blue) and independent quoll detections (grey) across all survey periods at (**a**) the Fragmented site (n = 12 surveys) and (**b**) the Intact site (n = 7 surveys). Dotted lines indicates the standard error.

**Figure 4:**
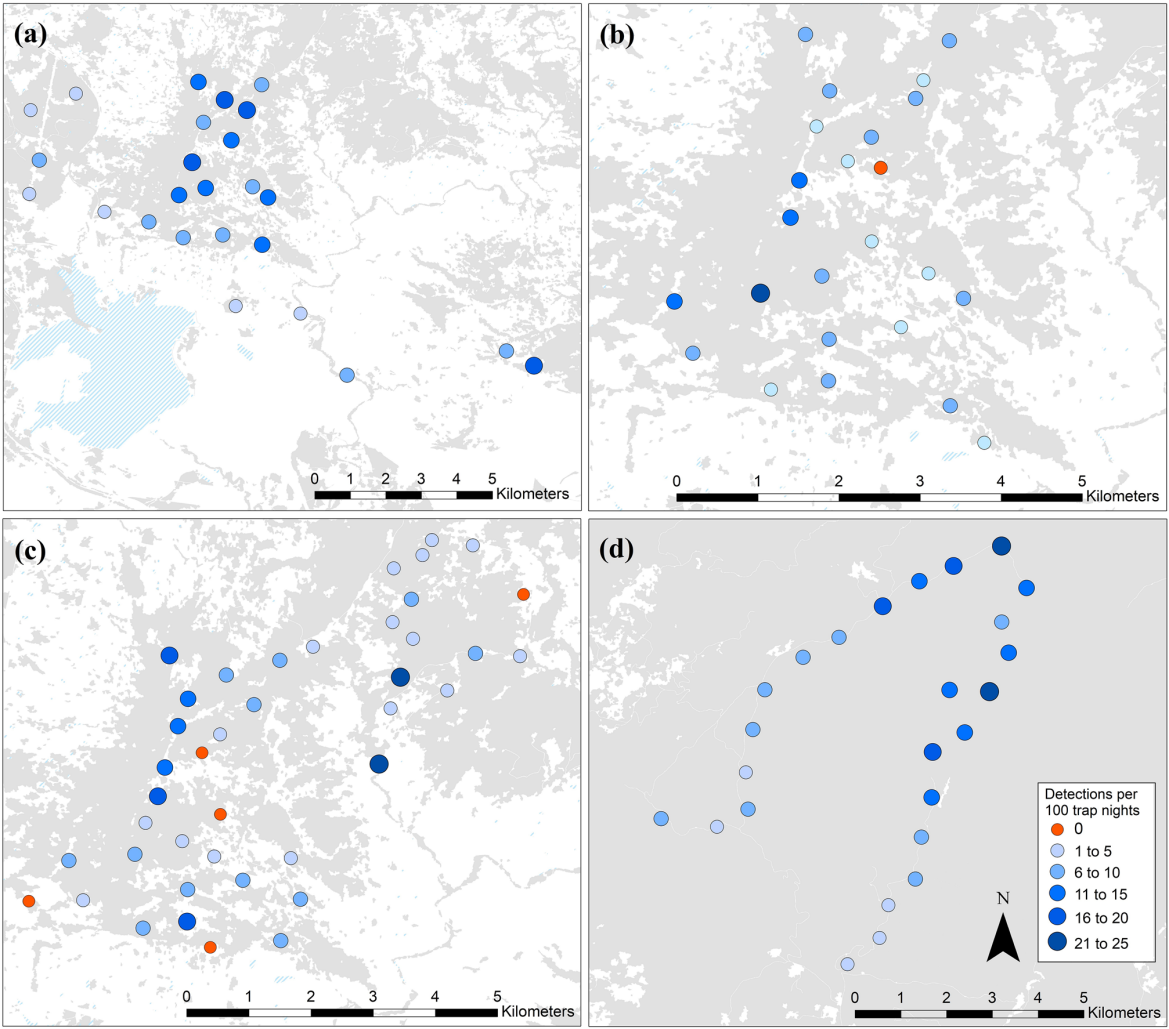
The number of independent quoll detections per 100 camera trap nights at each camera location (and across all surveys) for the Fragmented site: (**a**) Study 1, (**b**) Study 2, and (**c**) Study 3; and (**d**) the Intact site. This figure was created using ArcGIS version 10.4.1 (www.esri.com).

At both sites, cumulative quoll detections increased almost linearly with time across all 42-night survey periods (Fig. 3). On average, 90% of individual quolls were detected within 27 nights at the Fragmented site (Fig. 3a) and within 28 nights at the Intact site (Fig. 3b). This indicates that while most individual quoll were detected within the first ~ 4 weeks, quoll detections (of mostly the same individuals) continued at a consistent rate throughout the survey duration. At the Fragmented site, quolls were detected at almost all camera locations (Fig. 4a–c). At the Intact site, quolls were detected at all camera locations (Fig. 4d).

### Estimated quoll density

For each survey period, model comparison revealed substantial support for the top-ranked model (ΔAICc > 2) when compared to all other competing models (Supplementary Table S1 and S2). The ‘site-specific learned response’ model (bk) was the most frequently supported model at both the Fragmented site (7 out of 12 surveys; Table 3) and Intact site (4 out of 7 surveys; Table 4). There was typically support for the ‘bk’ model in surveys with high quoll detections, indicating that individual quolls tended to be re-detected at the same camera trap site.

**Table 3:** Summary of spatial capture-recapture quoll density estimates (quolls per km^2^), 95% confidence intervals (C.I.), and associated detection data for spotted-tailed quolls (*Dasyurus maculatus*) at the Fragmented site. Density estimates and associated Akaike Information Criterion values corrected for small sample sizes (AICc), are shown for the best fitting model for each survey: bk = site-specific learned response; T = time; and 0 = null model (see Table S1 for full details).

Survey dates	No. of quolls	No. of detections	Quolls (%) re-detected	Quolls (%) at ≥ 2 sites	Best model	AICc	Quolls per km^2^ (95% C.I.)
**Study 1**							
Jul–Aug 2018	18	73	15 (83%)	11 (61%)	bk	722.85	0.23 (0.14–0.40)
Oct–Nov 2018	14	42	7 (50%)	5 (36%)	T	419.93	0.21 (0.12–0.38)
Nov–Dec 2018	15	47	12 (80%)	5 (33%)	T	427.98	0.51 (0.29–0.90)
Feb–Mar 2019	14	40	10 (71%)	7 (50%)	0	439.08	0.33 (0.18–0.59)
Apr–May 2019	28	82	21 (75%)	17 (61%)	bk	868.12	0.42 (0.27–0.67)
Jun–Jul 2019	30	187	24 (80%)	22 (73%)	bk	1717.00	0.29 (0.19–0.44)
Aug–Sep 2019	19	121	16 (84%)	12 (63%)	bk	1129.31	0.18 (0.12–0.29)
**Study 2**							
Oct–Dec 2019	11	74	10 (91%)	8 (73%)	T	738.62	0.24 (0.13–0.45)
Mar–Apr 2020	14	60	10 (71%)	9 (64%)	T	641.55	0.35 (0.19–0.64)
**Study 3**							
May–Jun 2020	26	145	21 (81%)	18 (69%)	bk	1594.33	0.27 (0.18–0.43)
Aug–Sep 2020	15	127	13 (87%)	11 (73%)	bk	1327.46	0.13 (0.08–0.22)
Nov–Dec 2020	18	48	11 (61%)	6 (33%)	bk	497.27	0.66 (0.32–1.36)

**Table 4:** Summary of spatial capture-recapture quoll density estimates (quolls per km^2^), 95% confidence intervals (C.I.), and associated detection data for spotted-tailed quolls (*Dasyurus maculatus*) at the Intact site. Density estimates and associated Akaike Information Criterion values corrected for small sample sizes (AICc), are shown for the best fitting model for each survey: b = learned response; bk = site-specific learned response; and T = time (see Table S2 for full details).

Survey dates	No. of quolls	No. of detections	Quolls (%) re-detected	Quolls (%) at ≥ 2 sites	Best model	AICc	Quolls per km^2^ (95% C.I.)
Aug–Sep 2019	18	71	14 (78%)	12 (67%)	b	691.95	0.40 (0.21–0.75)
Jan–Feb 2020	24	49	15 (63%)	9 (38%)	T	530.37	0.47 (0.28–0.76)
Apr–May 2020	26	99	19 (73%)	16 (62%)	bk	971.11	0.37 (0.24–0.56)
Jul–Aug 2020	31	202	22 (71%)	18 (58%)	bk	1678.98	0.35 (0.24–0.50)
Oct–Nov 2020	18	77	16 (89%)	12 (67%)	T	725.63	0.28 (0.17–0.45)
Jan–Mar 2021	22	59	14 (64%)	9 (41%)	bk	605.27	0.48 (0.27–0.86)
May–Jun 2021	30	158	22 (73%)	17 (57%)	bk	1451.01	0.35 (0.24–0.51)

At the Fragmented site, estimated quoll density varied across survey periods, ranging from 0.13 (95% CI: 0.08–0.22) to 0.66 (95% CI: 0.32–1.36) quolls per km^2^, with an average of 0.32 ± 0.04 (s.e.) quolls per km^2^ (Table 3). Density at the Intact site was similar to the Fragmented site but was more consistent across survey periods, ranging from 0.28 (95% CI: 0.17–0.45) to 0.48 (95% CI: 0.27–0.86) quolls per km^2^, with a slightly higher average of 0.39 ± 0.03 (s.e.) quolls per km^2^ (Table 4). Estimated quoll densities appeared to increase during the Nov-Dec surveys at the Fragmented site (Table 3), and during surveys between Jan-Mar at the intact site (Table 4), although these estimates had large confidence intervals, low detections, and fewer quolls with spatial re-detections. In surveys where detections and the number of quolls with spatial re-detections were higher, density estimates were generally lower but were typically more precise (narrower confidence intervals; Tables 3 and 4). This was particularly evident during Study 3 at the Fragmented site, where estimated quoll density during the Nov-Dec 2020 survey was substantially higher, but with much lower precision and fewer detections when compared to the May-Jun 2020 and Aug-Sep 2020 surveys (Table 3).

## Discussion

This study is the first to compare quoll densities between fragmented and intact forested landscapes. Using SCR models, we determined that quoll density estimates were comparable between the two sites, suggesting that fragmented habitats have the capacity to support quoll populations densities equal to intact landscapes. This highlights the importance of retaining and protecting forest fragments for the conservation of endangered quolls in Australia.

There are several possible explanations for the observed similarity in quoll densities across the fragmented and intact habitats. First, the apparent low density of foxes at our fragmented site^23^ might have allowed quolls to reach densities similar to those attainable in fox-free or intact forested habitats. Indeed, our estimated densities of 0.13–0.66 quolls per km^2^ at the fragmented site were similar to densities of 0.20–0.70 quolls per km^2^ observed by Hamer et al.^41^ in a fox-free fragmented agricultural landscape in Tasmania. On mainland Australia, quolls are considered vulnerable to negative interactions from foxes^42,43^, with exploitation and interference competition likely increased in fragmented landscapes^44,45^. However, this risk would be minimised at sites with few or no foxes. Fox detections at our intact site were low, with only three detections from 17,690 camera trap nights. Density estimates of 0.28–0.66 quolls per km^2^ at this site were similar to observed estimates of 0.10–0.50 quolls per km^2^ in an intact, forested landscape in north-eastern New South Wales, where foxes were rare^46^. Therefore, quolls may be able to equally occur in fragmented and intact forested habitats if fox density is below a certain threshold. Future research should investigate whether quoll densities decline across sites supporting a gradient of increasing fox densities.

Second, fragmented landscapes may provide sufficient habitat and prey resources to support quoll densities similar to intact forested landscapes. For example, forest fragments provide suitable den sites and refugia from other predators for long-tailed weasels (*Mustela frenata*) in agricultural landscapes in America. Likewise, leopard cats (*Prionailurus bengalensis*) exploit abundant rodent pests in landscapes fragmented by oil palm plantations in South-East Asia^47–49^. On mainland Australia, quolls are generally associated with contiguous forest comprising an abundance of hollow-bearing trees, logs, and burrows, which provide suitable den sites and support high prey densities^19,50,51^. Similarly, the fragmented site has an abundance of large boulders and rocky outcrops which also offer suitable den sites for quolls^20,50^, and may also support high densities of supplemental prey species such as invasive European hares (*Lepus europaeus*) and rodents, which were frequently detected on the cameras. It is possible that the fragmented site provides sufficient resources that encompass typical quoll home range size and can therefore sustain quoll densities similar to the intact site. However, there is likely a threshold level of habitat fragmentation for which quolls can tolerate before resources become insufficient and densities decline. Further research should therefore investigate quoll densities along a gradient of increasingly fragmented landscapes, to better understand how different levels of habitat fragmentation impact quoll density.

Third, the quoll population in the fragmented site might represent a ‘sink’ population, with the nearby, higher quality intact site representing a ‘source’ population which supplements quoll losses at the lower quality fragmented site. In fragmented landscapes, sink populations can persist in habitat fragments and exist within a larger metapopulation^52^. For quoll populations in these habitat fragments, mortality may exceed reproduction, and therefore rely on immigrants from populations in good quality source habitats where reproduction exceeds mortality^53^. Live-trapping data conducted during our study suggests that the fragmented site’s population was comprised mostly of younger quolls, compared to the intact site. It is possible that while the fragmented site can support similar quoll densities, it serves as a sink population where quolls die young and are rapidly replaced by immigrants from nearby populations. Therefore, the replacement of quolls from source populations could be masking negative impacts of habitat fragmentation on the population size. Future research should investigate whether quolls in the fragmented site are genetically similar to quolls in the nearby intact forested site, to determine if these populations are isolated or constitute a metapopulation.

Although density estimates were similar between the two sites, there were some inconsistencies between survey periods within each site including estimates with varying degrees of precision. Surveys that yielded a higher number of detections and spatial re-detections generally resulted in lower but more precise density estimates. For both sites, quoll detections were highest for surveys undertaken between May and August, and were often associated with narrower confidence intervals than other surveys. These survey periods coincided with the quolls’ annual breeding and immediate post-breeding periods^15^. During these times, quolls are most active due to the immigration of transient males from surrounding areas^46^, thereby increasing the likelihood of detection at the study site. This was also reflected in a higher number of individual quolls and detections, though surveys during this period generally yielded lower density estimates. In contrast, density estimates were generally higher during surveys undertaken between October and February which coincides with when juveniles typically emerge from their natal dens and disperse^46,54^. However, these estimates were associated with large confidence intervals and fewer quoll detections and spatial re-detections. Similar results were found in Schmidt, et al.^55^ where overly high density estimates of black bears (*Ursus americanus*) were associated with fewer individuals and relatively low spatial re-captures, suggesting inflation of density estimates. Spatial re-detections are particularly important for reducing inflated estimates, and surveys which detected fewer individuals with spatial re-detections may result in less precise density estimates.

Our study highlights some of the potential challenges in obtaining reliable density estimates for carnivores, even when reliable individual identification is achievable. SCR models offer an advantage over conventional capture-recapture methods by eliminating the need to estimate the size of the sample area, but instead requires sufficient spatial re-detections of individuals^28^. White^56^ recommends a minimum of 20 individuals to obtain precise population estimates for conventional capture-recapture models. However, this is not always achievable especially when attempting to survey low density, wide ranging carnivores^57^. Therefore, SCR surveys need to maximise the spatial re-detectability of individuals^58^. This might be achieved by deploying more cameras across a wider area^59^, increasing survey duration, or surveying during the time of the year when the animal is most active^60,61^. Conversely, consideration also needs to be given to the requirement for population closure^62^. In our study for instance, transient male quolls and independent juveniles may be entering or leaving the population during various times of the year. Therefore, a compromise is needed between survey length being long enough to obtain sufficient data, but also short enough so that the assumption of closure is less likely to be violated due to transient individuals^37^.

Understanding population density in different landscapes is critical for developing conservation and management strategies for mesopredator species. While quoll research has predominately been conducted in intact contiguous forest, our results suggest that quolls can equally persist at similar densities in fragmented habitats, highlighting the importance of forest fragments for quoll conservation. The importance of forest fragments in supporting mesopredator populations has similarly been documented for pine martens^63^, leopard cats^64^ and güiña^14^. Although quolls can persist and potentially thrive in fragmented Australian landscapes, further research is needed to determine associated limiting factors such as prey availability, habitat resources and genetic connectivity to proximal contiguous forests, to better inform appropriate conservation and management of this endangered species.

## Supplementary Information

Supplementary Information.

## Data Availability

The data used in this paper are available from the corresponding author on reasonable request.
