# Supplementary material for: Density estimates reveal that fragmented landscapes provide important habitat for conserving an endangered mesopredator, the spotted-tailed quoll
Source: Sci Rep. 2022 Jul 25;12:12688. doi: 10.1038/s41598-022-16982-x (PMC9314389; doi:10.1038/s41598-022-16982-x)
Supplement: Supplementary file 1 — Supplementary Information. [file 41598_2022_16982_MOESM1_ESM.docx]

Tagging summary (using ExifPro and PowerPoint):

1. Tag image sequence as ‘non-identifiable’ if there are no clear images of the quolls lateral sides, otherwise proceed to step 2.

2. Determine if image sequence includes clear images of one lateral side, or both lateral sides.

3. Tag images with either a pre-existing quoll label (if images match) or with a new label.

4. Add example images of lateral spot patterns to corresponding PowerPoint slide for profiled quoll.

5. Repeat process until all images are tagged.

BOTH

ONE

**4(a).**
This individual quoll profile is now complete. Additional lateral images can be added as identification progresses

COMPLETE

NO

**3(a).**
Create a new individual quoll label (e.g. Q001)

YES

NO

YES

**2.**Does the image sequence include images of **one** lateral side, or **both** lateral sides?

**3(b).**
Create a new individual quoll label (e.g. Q003). Replace the old tag with the new tag

**2(a).**
Do lateral spot patterns match with a pre-existing quoll profile (complete or semi-complete)?

SEMI-COMPLETE

**1.**
Does the image sequence show clear spot patterns of either/both of the quolls lateral side(s)?

**3(c).**
Tag image sequence to match pre-existing quoll label (e.g. Q002)

**3(d).**
Create a new individual quoll label, indicating that only **one-side** of the quoll has been profiled (e.g. L001 for left-hand side).

**3(e).**
Tag image sequence to match pre-existing quoll label (e.g. L001)

**4(b).**
This individual quoll profile is semi-complete. (e.g. left or right side only). Check images with new complete profiles as identification progresses

**1(a).**
Tag image sequence as ‘non-identifiable’
(e.g. NONID)

**2(b).**
Do lateral spot patterns match with a semi-complete quoll profile?

NO

YES

**Figure S1.** Flow-chart for tagging individual quolls using ExifPro and PowerPoint.


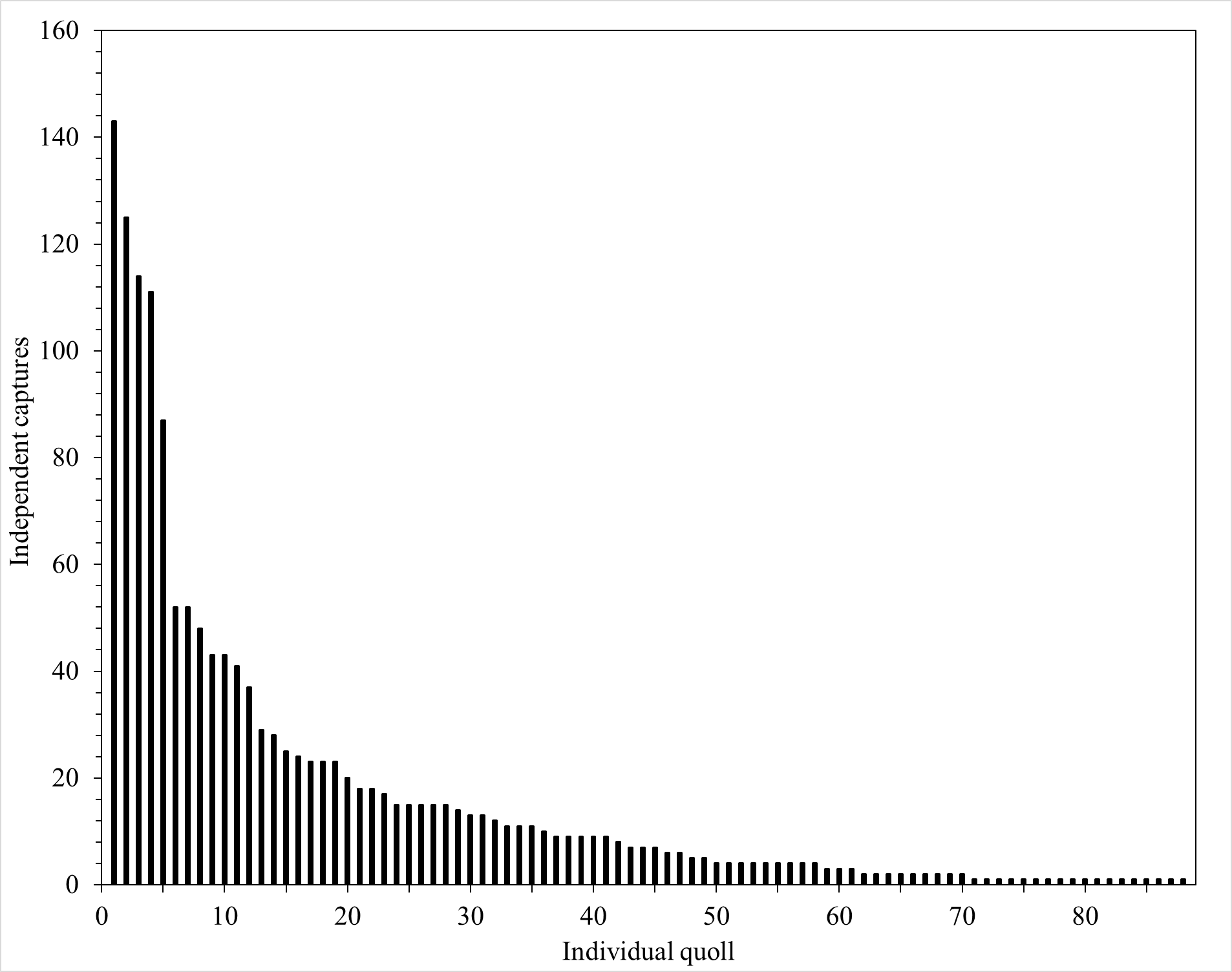


**Figure S2.** The number of times each individual quoll was detected at the Fragmented site. Each detection is an ‘independent event’ (≥10 mins between subsequent image sequences).

**
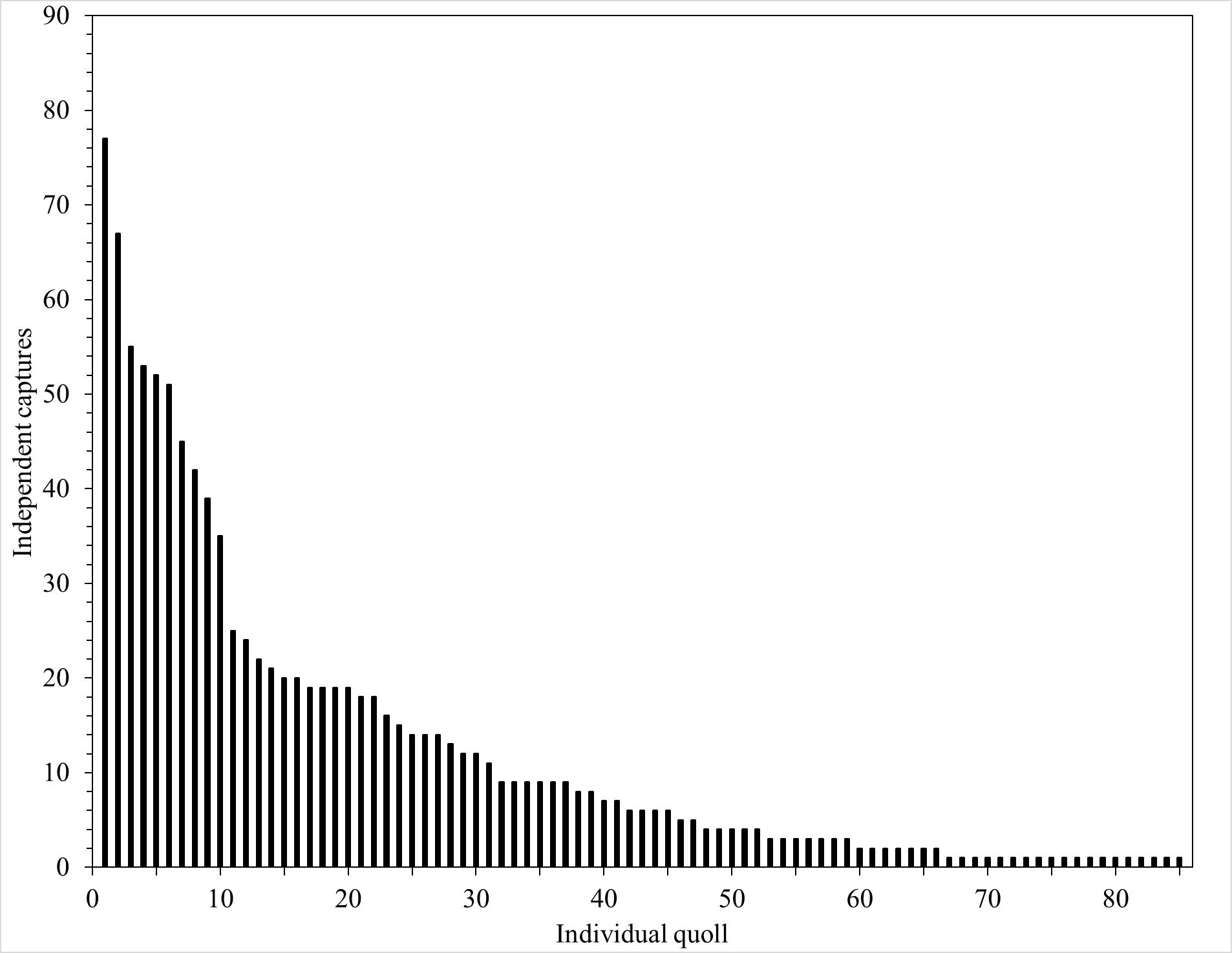
**

**Figure S3.** The number of times each individual quoll was detected at the Intact site. Each detection is an ‘independent event’ (≥10 mins between subsequent image sequences).

**Table S1.** Summary of spatial capture-recapture model outputs for each survey for the Fragmented site. K = total number of parameters; LL = log-likelihood; AICc = Akaike Information Criterion corrected for small sample size; ΔAICc = delta AICc; AIC *wt* = AICc weight.

| **Jul-Aug 2018** | | | | | |
| --- | --- | --- | --- | --- | --- |
| **Model** | **K** | **LL** | **AICc** | **ΔAICc** | **AICc *wt*** |
| bk | 4 | -355.89 | 722.85 | 0.00 | 1.00 |
| 0 | 3 | -364.00 | 735.72 | 12.87 | 0.00 |
| T | 4 | -362.67 | 736.42 | 13.57 | 0.00 |
| Bk | 4 | -363.21 | 737.50 | 14.65 | 0.00 |
| b | 4 | -363.53 | 738.14 | 15.29 | 0.00 |
| B | 4 | -363.94 | 738.97 | 16.12 | 0.00 |
| **Oct-Nov 2018** | | | | | |
| **Model** | **K** | **LL** | **AICc** | **ΔAICc** | **AICc *wt*** |
| T | 4 | -203.74 | 419.93 | 0.00 | 0.98 |
| B | 4 | -208.14 | 428.73 | 8.80 | 0.01 |
| 0 | 3 | -210.68 | 429.76 | 9.83 | 0.01 |
| bk | 4 | -209.37 | 431.18 | 11.25 | 0.00 |
| Bk | 4 | -209.63 | 431.71 | 11.78 | 0.00 |
| b | 4 | -210.68 | 433.80 | 13.87 | 0.00 |
| **Nov-Dec 2018** | | | | | |
| **Model** | **K** | **LL** | **AICc** | **ΔAICc** | **AICc *wt*** |
| T | 4 | -207.99 | 427.97 | 0.00 | 0.95 |
| bk | 4 | -211.20 | 434.41 | 6.44 | 0.04 |
| b | 4 | -212.84 | 437.69 | 9.72 | 0.01 |
| 0 | 3 | -215.19 | 438.56 | 10.59 | 0.00 |
| B | 4 | -214.39 | 440.78 | 12.81 | 0.00 |
| Bk | 4 | -214.76 | 441.51 | 13.54 | 0.00 |
| **Feb-Mar 2019** | | | | | |
| **Model** | **K** | **LL** | **AICc** | **ΔAICc** | **AICc *wt*** |
| 0 | 3 | -215.34 | 439.08 | 0.00 | 0.45 |
| T | 4 | -214.45 | 441.35 | 2.28 | 0.14 |
| b | 4 | -214.61 | 441.66 | 2.58 | 0.12 |
| bk | 4 | -214.63 | 441.70 | 2.62 | 0.12 |
| B | 4 | -214.86 | 442.17 | 3.10 | 0.10 |
| Bk | 4 | -215.31 | 443.06 | 3.98 | 0.06 |
| **Apr-May 2019** | | | | | |
| **Model** | **K** | **LL** | **AICc** | **ΔAICc** | **AICc *wt*** |
| bk | 4 | -429.19 | 868.12 | 0.00 | 0.97 |
| T | 4 | -432.67 | 875.07 | 6.96 | 0.03 |
| 0 | 3 | -436.03 | 879.06 | 10.95 | 0.00 |
| Bk | 4 | -435.81 | 881.36 | 13.24 | 0.00 |
| B | 4 | -435.92 | 881.58 | 13.47 | 0.00 |
| b | 4 | -435.99 | 881.73 | 13.61 | 0.00 |
| **Jun-Jul 2019** | | | | | |
| **Model** | **K** | **LL** | **AICc** | **ΔAICc** | **AICc *wt*** |
| bk | 4 | -853.70 | 1717.00 | 0.00 | 1.00 |
| Bk | 4 | -868.44 | 1746.48 | 29.48 | 0.00 |
| B | 4 | -875.21 | 1760.02 | 43.02 | 0.00 |
| b | 4 | -881.22 | 1772.04 | 55.03 | 0.00 |
| 0 | 3 | -883.66 | 1774.25 | 57.25 | 0.00 |
| T | 4 | -883.11 | 1775.81 | 58.81 | 0.00 |
| **Aug-Sep 2019** | | | | | |
| **Model** | **K** | **LL** | **AICc** | **ΔAICc** | **AICc *wt*** |
| bk | 4 | -559.23 | 1129.31 | 0.00 | 0.56 |
| Bk | 4 | -560.42 | 1131.70 | 2.39 | 0.17 |
| T | 4 | -560.59 | 1132.03 | 2.72 | 0.14 |
| 0 | 3 | -562.82 | 1133.24 | 3.93 | 0.08 |
| b | 4 | -562.15 | 1135.15 | 5.84 | 0.03 |
| B | 4 | -562.32 | 1135.49 | 6.18 | 0.03 |
| **Table S1** continued. | | | | | |
| **Oct-Dec 2019** | | | | | |
| **Model** | **K** | **LL** | **AICc** | **ΔAICc** | **AICc *wt*** |
| T | 4 | -361.98 | 738.62 | 0.00 | 1.00 |
| B | 4 | -378.74 | 772.14 | 33.52 | 0.00 |
| 0 | 3 | -382.24 | 773.91 | 35.29 | 0.00 |
| b | 4 | -380.09 | 774.84 | 36.22 | 0.00 |
| Bk | 4 | -380.97 | 776.60 | 37.98 | 0.00 |
| bk | 4 | -382.04 | 778.75 | 40.13 | 0.00 |
| **Mar-Apr 2020** | | | | | |
| **Model** | **K** | **LL** | **AICc** | **ΔAICc** | **AICc *wt*** |
| T | 4 | -314.6 | 641.5 | 0.0 | 1.0 |
| B | 4 | -321.5 | 655.4 | 13.9 | 0.0 |
| b | 4 | -324.9 | 662.2 | 20.7 | 0.0 |
| 0 | 3 | -327.8 | 664.0 | 22.4 | 0.0 |
| bk | 4 | -326.2 | 664.9 | 23.4 | 0.0 |
| Bk | 4 | -327.4 | 667.2 | 25.6 | 0.0 |
| **May-Jun 2020** | | | | | |
| **Model** | **K** | **LL** | **AICc** | **ΔAICc** | **AICc *wt*** |
| bk | 4 | -792.21 | 1594.33 | 0.00 | 1.00 |
| Bk | 4 | -817.27 | 1644.44 | 50.11 | 0.00 |
| T | 4 | -820.04 | 1649.99 | 55.66 | 0.00 |
| B | 4 | -822.46 | 1654.82 | 60.49 | 0.00 |
| 0 | 3 | -824.81 | 1656.70 | 62.37 | 0.00 |
| b | 4 | -824.20 | 1658.30 | 63.97 | 0.00 |
| **Aug-Sep 2020** | | | | | |
| **Model** | **K** | **LL** | **AICc** | **ΔAICc** | **AICc *wt*** |
| bk | 4 | -657.78 | 1327.56 | 0.00 | 1.00 |
| T | 4 | -670.66 | 1353.31 | 25.75 | 0.00 |
| 0 | 3 | -673.98 | 1356.15 | 28.59 | 0.00 |
| Bk | 4 | -672.95 | 1357.90 | 30.34 | 0.00 |
| b | 4 | -673.31 | 1358.63 | 31.07 | 0.00 |
| B | 4 | -673.39 | 1358.78 | 31.22 | 0.00 |
| **Nov-Dec 2020** | | | | | |
| **Model** | **K** | **LL** | **AICc** | **ΔAICc** | **AICc *wt*** |
| bk | 4 | -243.09 | 497.27 | 0.00 | 1.00 |
| b | 4 | -269.07 | 549.21 | 51.95 | 0.00 |
| Bk | 4 | -271.56 | 554.19 | 56.93 | 0.00 |
| T | 4 | -272.88 | 556.84 | 59.58 | 0.00 |
| 0 | 3 | -276.12 | 559.95 | 62.68 | 0.00 |
| B | 4 | -275.49 | 562.06 | 64.79 | 0.00 |

**Table S2.** Summary of spatial capture-recapture model outputs for each survey for the Intact site. K = total number of parameters; LL = log-likelihood; AICc = Akaike Information Criterion corrected for small sample size; ΔAICc = delta AICc; AIC *wt* = AICc weight.

| **Aug-Sep 2019** | | | | | |
| --- | --- | --- | --- | --- | --- |
| **Model** | **K** | **LL** | **AICc** | **ΔAICc** | **AICc *wt*** |
| b | 4 | -340.44 | 691.95 | 0.00 | 0.60 |
| bk | 4 | -341.46 | 694.00 | 2.05 | 0.22 |
| 0 | 3 | -344.07 | 695.85 | 3.90 | 0.09 |
| B | 4 | -342.79 | 696.67 | 4.72 | 0.06 |
| Bk | 4 | -343.59 | 698.26 | 6.31 | 0.03 |
| T | 4 | -344.06 | 699.20 | 7.25 | 0.02 |
| **Jan-Feb 2020** | | | | | |
| **Model** | **K** | **LL** | **AICc** | **ΔAICc** | **AICc *wt*** |
| T | 4 | -260.13 | 530.37 | 0.00 | 0.84 |
| b | 4 | -262.55 | 535.21 | 4.85 | 0.07 |
| bk | 4 | -262.61 | 535.33 | 4.96 | 0.07 |
| 0 | 3 | -266.16 | 539.52 | 9.15 | 0.01 |
| Bk | 4 | -264.95 | 540.01 | 9.64 | 0.01 |
| B | 4 | -265.88 | 541.87 | 11.50 | 0.00 |
| **Apr-May 2020** | | | | | |
| **Model** | **K** | **LL** | **AICc** | **ΔAICc** | **AICc *wt*** |
| bk | 4 | -480.60 | 971.11 | 0.00 | 0.97 |
| b | 4 | -484.10 | 978.11 | 7.01 | 0.03 |
| B | 4 | -487.18 | 984.26 | 13.16 | 0.00 |
| Bk | 4 | -487.48 | 984.86 | 13.76 | 0.00 |
| T | 4 | -487.52 | 984.94 | 13.84 | 0.00 |
| 0 | 3 | -490.79 | 988.67 | 17.56 | 0.00 |
| **Jul-Aug 2020** | | | | | |
| **Model** | **K** | **LL** | **AICc** | **ΔAICc** | **AICc *wt*** |
| bk | 4 | -834.72 | 1678.98 | 0.00 | 1.00 |
| Bk | 4 | -854.16 | 1717.85 | 38.87 | 0.00 |
| b | 4 | -854.74 | 1719.03 | 40.05 | 0.00 |
| B | 4 | -855.52 | 1720.57 | 41.59 | 0.00 |
| 0 | 3 | -857.93 | 1722.75 | 43.77 | 0.00 |
| T | 4 | -857.10 | 1723.73 | 44.75 | 0.00 |
| **Oct-Nov 2020** | | | | | |
| **Model** | **K** | **LL** | **AICc** | **ΔAICc** | **AICc *wt*** |
| T | 4 | -357.28 | 725.63 | 0.00 | 1.00 |
| 0 | 3 | -365.57 | 738.85 | 13.22 | 0.00 |
| b | 4 | -365.19 | 741.45 | 15.83 | 0.00 |
| Bk | 4 | -365.38 | 741.84 | 16.21 | 0.00 |
| bk | 4 | -365.44 | 741.95 | 16.32 | 0.00 |
| B | 4 | -365.57 | 742.21 | 16.58 | 0.00 |
| **Jan-Mar 2021** | | | | | |
| **Model** | **K** | **LL** | **AICc** | **ΔAICc** | **AICc *wt*** |
| bk | 4 | -297.46 | 605.27 | 0.00 | 0.91 |
| T | 4 | -299.81 | 609.96 | 4.70 | 0.09 |
| b | 4 | -303.47 | 617.29 | 12.02 | 0.00 |
| 0 | 3 | -306.92 | 621.17 | 15.90 | 0.00 |
| Bk | 4 | -306.69 | 623.73 | 18.46 | 0.00 |
| B | 4 | -306.90 | 624.14 | 18.88 | 0.00 |
| **May-Jun 2021** | | | | | |
| **Model** | **K** | **LL** | **AICc** | **ΔAICc** | **AICc *wt*** |
| bk | 4 | -720.71 | 1451.01 | 0.00 | 1.00 |
| B | 4 | -730.44 | 1470.48 | 19.47 | 0.00 |
| Bk | 4 | -734.64 | 1478.87 | 27.86 | 0.00 |
| b | 4 | -734.87 | 1479.34 | 28.33 | 0.00 |
| 0 | 3 | -737.01 | 1480.94 | 29.93 | 0.00 |
| T | 4 | -736.21 | 1482.03 | 31.01 | 0.00 |
